# Cancer Multidisciplinary Teams in Africa: A Narrative Review of Their Role and Availability

**DOI:** 10.7759/cureus.84553

**Published:** 2025-05-21

**Authors:** Quadri A Sanni, Abdulkadir Mohamed, James Tsejime, Ejike H Anyaegbu, Jubril O Anifowose, Kay'ien Wong, Mia V Mechelen

**Affiliations:** 1 Department of Urology, South Warwickshire University NHS Foundation Trust, Warwick, GBR; 2 Department of Surgery, South Warwickshire University NHS Foundation Trust, Warwick, GBR; 3 Department of Emergency Medicine, South Warwickshire University NHS Foundation Trust, Warwick, GBR; 4 Department of Medicine, Southend University Hospital NHS Foundation Trust, Essex, GBR; 5 General Medicine, Caterham School, Caterham, GBR; 6 General Medicine, The National Mathematics and Science College, Coventry, GBR

**Keywords:** cancer center, healthcare outcomes, multidisciplinary discussion, multidisciplinary team, oncology care, sub-saharan africa

## Abstract

Multidisciplinary teams (MDTs) play a pivotal role in the care of cancer patients. With a progressive rise in the incidence of cancer diagnoses in the African subcontinent, there is an expectation that oncologic care should be standardized and fairly available, and central to this care is the establishment of a cancer MDT. This study aimed to explore the availability and role of oncologic MDTs in various centers in the African subcontinent. Data on Cancer MDTs were searched using keywords on PubMed, African Journal Online, and Google Scholar. Other relevant searches were conducted using the reference list of primary studies. There has been a 34% increase in the prevalence of MDT meetings among institutions in sub-Saharan Africa, spanning all regions of Africa, based on a survey conducted by the African Organisation for Research and Treatment in Cancer network in 2013 and repeated in 2021. Although there is an increase in the prevalence of MDT meetings in sub-Saharan Africa, spanning all regions of Africa, the mortality rate of cancer patients remains disproportionately high, with a mortality rate higher than that of Europe. Several centers across African countries do not have access to functioning oncologic MDTs, and those with access do have members frequently missing. Several factors mitigate against the establishment of cancer MDTs in Africa, including the lack of specialists, motivation of the healthcare staff, poor financial remuneration, and government policies. To ensure improved cancer care in Africa, efforts should be made to involve key stakeholders, including healthcare professionals, the government, and the private sector.

## Introduction and background

The World Health Organization (WHO) has reported that the incidence of non-communicable diseases is on the rise, with cancer diagnoses at the forefront of these diseases [1]. In low and middle-income countries, 60% of worldwide cancer patients are diagnosed [2], and a substantial proportion of such patients, many of whom reside in Africa, are potentially prevented from obtaining gold-standard, evidence-based care that is afforded in the Western world [3,4]. Central to this gold-standard care is the use of a multidisciplinary team (MDT) [5,6]. The concept of MDT can be traced back to the time of Hippocrates [7]. During the time of the First Crusade, many pilgrims and warriors were killed, and a directive was issued by the then Pope to create facilities for medical care. This led to the creation of the first organized MDT [7]. MDT is crucial in personalized cancer management, and usually involves surgeons, clinicians, nurses, radiologists, and pathologists to manage complex cases at a given time and frequency, either physically or virtually, virtual being a commonly used modality in remote areas devoid of specialist input [8-10]. Management of oncologic patients without contribution from MDTs can lead to inaccurately staged and treated cancer, challenges with diagnosis and management, and does not usually conform to evidence-based practice, leading to poor outcomes [11-14]. A study from the United States demonstrated the risk of not having an MDT, showing how for gynecological cancers, MDT input changed management in over a third of cases, along with redefinition of the cancer, which impacted prognostics and further management [15]. Similar findings have been published from New Zealand [16]. Such misdiagnoses can also be devastating for families, as well as those who rely on misinformed prognostic information.

Further, MDTs are empirical in complex cases where guidelines do not align with the clinical case at hand and for which there is insufficient evidence base or when guidelines have been exhausted [10,11,17]. MDTs can also be involved in the development of new guidelines [18]. In the Western world, such teams meet weekly to discuss cases as they evolve; however, such regularity and attendance may be deficient in developing countries, even if an MDT is present [19]. This creates a situation in which healthcare services have a substantial case burden but irregular MDT, leading to delayed treatment and worse patient outcomes [20]. In addition, even when such a service exists in the developing world, there are local and national inconsistencies [10], which add to socioeconomic problems, such as inequity and healthcare disparities. There has been some progress to remedy this concern in other developing countries, such as in India, through virtual MDT centrally organized by the government; however, political issues in several countries in Africa can limit this [10]. Other commonly cited reasons for failings of proper MDT implementation include fragmented care [20] and insufficient human resources [18,21].

However, there are examples of substantial changes to patient outcomes when an MDT operates in an African healthcare system. For example, the implementation of an MDT for Wilms tumour in children in a Nigerian tertiary hospital [22] led to a 13% increase in treatment completion, which highlights the importance of counselling which can be delivered by nurses, ensuring the compliance of treatment and subsequent better outcomes (five-year survival increasing from 31.48% to 75%). It also highlights the need for collaboration between physicians to optimize treatment, e.g., in terms of compromising for side effects, allowing for better treatment uptake [22]. Despite such positive examples in the literature, the economics of MDT implementation have been questionable. Some studies have commented on MDT being an expensive resource that does not justify its benefit, arguing that it is only effective in a select group of patients, particularly fit and young patients with early disease [23,24]. However, there are African studies that prove the cost-effectiveness of MDT for cancer care. For example, a study from Mozambique demonstrated the benefit in terms of quality-adjusted life years (QALYs), making the incremental cost of MDT implementation worthwhile [17]. There are also suggestions to further the cost-effectiveness of MDT through patient stratification and prioritization by the creation of distinct guidelines to guide case presentation [11,19].

In addition to the numerous studies delineating the substantive benefit to the patient being discussed at an MDT, there are also secondary benefits to an MDT improving future cancer care. It is commonly attended by more junior members of the team and can serve as an inadvertent teaching platform [10,19,25,26]. Interdisciplinary collaboration can also spark innovation in the form of novel clinical trials [10].

Furthermore, MDTs that cross national borders have proven to have benefits in knowledge transfer and mentorship between doctors. For example, the Edinburgh-Malawi cancer partnership and the Mayo (United States)-Kumasi, Ghana training partnership [18,21]. Inter-country collaboration between a developed and a developing healthcare system further allows African physicians to appreciate the benefits to patient care offered by MDTs, and subsequently inspire the development of an effective MDT in their healthcare system, with assistance from the developed healthcare system. Such collaborations also facilitate interactions outside of video meetings in the form of emails to discuss complex cases [21].

The objective of this narrative review is to highlight, through the use of the latest literature, the availability and purpose of cancer MDTs in Africa, focusing on the current state and insight among patients and clinicians, lessons learnt, and projections for the future.

## Review

Methodology

A search of the literature was conducted using keywords related to the cancer MDT on PubMed, Google Scholar, and African Journal Online. We included studies published in English or French that discussed the availability, structure, and impact of MDTs in cancer management within African countries, discussing the barriers and facilitators of MDT implementation. We excluded studies that focused on high-income countries, opinion pieces or editorials with no empirical data, and studies on non-cancer MDTs. The articles were then analyzed by the authors to ensure they conform to the inclusion criteria.

Overview of multidisciplinary teams in cancer care

In the context of cancer care, MDTs focus on patient diagnosis, treatment planning, and management to enhance clarity. MDT comprises individuals from different specialities who come together with a common goal, either to introduce a new project, redesign an existing project, or provide services or care differently [27]. There is no standardized format for organizing MDTs [27]. The level of integration can range from a single professional with continued responsibility for care drawing on other staff or services for input, to multiple professionals holding shared responsibility for the care of the services, potentially drawing on a much wider pool of services and professionals [3]. A cancer MDT consists of integrated healthcare professionals from different specialities working together to provide a comprehensive and individualized approach to the diagnosis, treatment, and management of cancer patients [28]. The purpose of the cancer MDT is to provide holistic care through an improved decision-making process via an integrated community of healthcare professionals. Core members of a cancer MDT, therefore, include medical oncologists, surgeons, radiation oncologists, radiologists, pathologists, oncology nurses, palliative care specialists, and the MDT coordinator [29].

Whereas a medical oncologist specializes in systemic treatments and manages adjuvant and palliative treatments on the cancer MDT, the surgeon performs tumor biopsies and resections and plans and executes curative and palliative surgeries [30]. The pathologist examines biopsies and provides histopathological reports. Pertinent to the pathologist’s role on the cancer MDT is to identify cancer type, grade, and molecular marker [30]. The radiologist interprets CT, MRI, and positron emission tomography (PET) scans, as well as plain radiographs, for the diagnosis and staging of cancer. The radiation oncologist specializes in radiotherapy planning and delivery and provides palliative versus curative intent radiotherapy [31,32]. Another important member of the cancer MDT is the oncology nurse. The oncology nurse acts as a liaison between the cancer patient and MDT, providing patient support, education, and symptom management [33]. The palliative care specialists form a crucial arm of the cancer MDT as they manage pain relief and provide symptom control and end-of-life care. The palliative care specialists help with the quality of life discussions [27,34]. The coordinator organizes the cancer MDT meetings, keeps a record of discussions, and ensures timely communication among members of the MDT.

Globally, MDT has functioned effectively in managing complex diseases such as cancer with improved outcomes [27]. This approach to patient care emerged in the mid-1980s, when the addition of chemotherapy to radiotherapy and/or surgery was proven to improve survival, and has grown to its current state [3]. The first functional units of the MDT created in Europe were the breast cancer treatment units formed within institutions and focused on the diagnosis, treatment, genetic counselling, psycho-social support, and research [28]. The roles of these breast cancer units were standardized in 2013, when the European Society of Breast Cancer Specialists published a consensus on the minimum requirements for the multidisciplinary management of breast cancers [29]. These guidelines were well-received by medical specialists, leading to the introduction of the present-day MDT in many European countries [30]. In the United Kingdom, MDT has become the cornerstone for cancer care, ensuring that patients receive the best input from various specialists to determine the most effective treatment strategies [35]. Therefore, MDT approaches to patient care are well established in Europe and enhance cancer care, highlighting improved outcomes with standardized treatment strategies.

While the above highlighted the effectiveness of cancer MDTs, it is important to note that MDTs have also been successful in managing other complex illnesses besides cancer.

In the United States, MDT approaches are integral to patient management, emphasizing collaborative care across various healthcare settings, and have gone through several stages to the present state, with several models and initiatives used to effectively implement the MDT approach to patient care [36]. The guided care model developed by experts at the Johns Hopkins Bloomberg School of Public Health assigns a registered nurse to work closely with physicians in primary care settings [37]. This nurse coordinates care for patients with multiple chronic conditions by conducting comprehensive assessments, creating care plans, monitoring health, and facilitating communication among healthcare providers [37].

Besides the above-mentioned models, the structured Interdisciplinary Bedside Rounds involve a structured process where the healthcare team, including physicians, nurses, and other allied healthcare professionals, conduct rounds at the patient’s bedside [38]. This approach fosters real-time collaboration, enhances communication, and ensures that care plans are tailored to patients’ needs [38]. The Rapid Response System is also an effective model designed to identify and respond to patients exhibiting early signs of clinical deterioration in non-intensive care units [39]. A multidisciplinary rapid response team, typically composed of critical care physicians, nurses, and respiratory therapists, intervenes promptly to prevent adverse events such as cardiac or respiratory arrest [39]. These models highlight the integral role of MDTs in advancing patient care in the United States, demonstrating the benefits and establishment of this approach to patient care.

The benefits of MDT in cancer care are multifactorial. The MDT approach to cancer care enhances treatment effectiveness, improves patient outcomes, and provides holistic support [40]. By integrating expertise from various specialists, MDT ensures that cancer patients receive personalized, well-coordinated, and high-quality care, improving their quality of life and survival rate [40]. A multidisciplinary approach integrates expertise from different specialities, thereby offering a faster and accurate diagnosis [40]. This is because the team can efficiently interpret test results, imaging, and pathology reports, leading to quicker diagnosis with a timely treatment initiation [32,34,40]. Besides an improved diagnosis, cancer MDT provides a comprehensive and personalized treatment plan [28,29,40]. Each cancer case is reviewed by a team of specialists who consider various factors such as tumor stage, genetic markers, and patient preferences when designing a treatment plan [30]. Personalized care, therefore, improves the effectiveness of treatment and enhances the quality of life.

Furthermore, cancer MDT ensures enhanced patient support and holistic care [29]. Composed of diverse members, it offers support services such as psychological counselling, palliative care, and social support. Patients receive emotional, social, and practical support, helping them cope with the challenges of cancer treatment [29,30,33,34]. MDT in cancer care results in improved communication and shared decision-making among healthcare providers and patients [41].

Lastly, integrating MDT approaches into the education of healthcare physicians offers enhanced collaborative skills, which foster effective communication and teamwork among diverse healthcare professionals, leading to improved patient outcomes and reduced adverse events [41,42]. Exposure to various specialities within the MDTs also enriches physicians’ understanding of different roles and responsibilities, promoting comprehensive patient care [29]. Incorporating MDT principles into medical education equips physicians with the skills necessary for modern, collaborative healthcare environments, ultimately benefiting practitioners and patients [28,42].

In summary, an MDT approach to cancer care brings together specialists from different fields to collaborate on patient diagnosis, treatment, and management. This approach improves patient outcomes by ensuring comprehensive, coordinated, and patient-centered care.

Current state of multidisciplinary teams in Africa

The uptake of MDT has been gradually increasing in Sub-Saharan Africa (SSA). A survey conducted through the African Organisation for Research and Treatment in Cancer (AORTIC) network in 2013 and repeated in 2021 showed a 34% increase in the prevalence of MDT meetings among institutions in SSA spanning all regions of Africa [43]. However, the mortality rate of breast cancer patients remains disproportionately high, with a mortality rate of 19.4/100,000 in breast cancer patients compared to 14.4/100,000 in Europe [44]. The difference in these figures is made even more stark when considered alongside the fact that the incidence rate of cancers is twice as high in developing countries compared to developed countries and that half of deaths from breast cancer come from SSA [45].

Nevertheless, the implementation of MDTs in SSA has proved to significantly increase survival in patients with cancer in Mozambique. This is highlighted by a decrease of 53% in the mortality rate among patients with early breast cancer [17]. An evidence-based list of characteristics of an effective MDT team highlights attendance, attitudes to team working, availability of technology and equipment, regularity of meetings, evidence-based guidelines, and organizational support [46]. Reports have shown that in 30 institutions across 21 SSA countries, 90% of institutions had weekly breast MDTs and consulted treatment guidelines, highlighting some of the core characteristics of an effective MDT, namely, regularity of the meeting and use of evidence-based guidelines. These reports did not ascertain the composition of the MDT meetings except that they are oncologist-led [43].

On the other hand, some of the characteristics that were lacking were primarily regarding the availability of technology and equipment, the absence of required specialty clinicians and nurses, and organizational support [43]. There are still wide disparities in the treatment of cancer between developing countries in SSA and developed countries. There is also a wide disparity between countries in SSA in terms of the equipment needed to provide anti-cancer care [43]. Overall, 28% of the countries in the study presented in the AORTIC survey had no recorded radiotherapy facilities, and, similarly, roughly 25% of institutions from the SSA countries in the survey reported frequent equipment downtimes [43]. Furthermore, government finance was absent in 36% of institutions, and, in 17% of institutions, the treatment cost was the complete responsibility of the patient. The biggest challenge facing MDT implementation in SSA is that most of the cancer cases present with advanced disease [43]. Hence, given the current implementation of MDT in SSA, there might also be a supply-demand mismatch between MDT provision and the number of patients whose cases require an MDT discussion, leading to further stress on a maturing system, which may be complicated by improper implementation.

Conversely, another study examining the MDT approach in Rwanda showed that adaptations to low resources in the form of an MDT-like approach are being applied where there is a paucity of some clinical disciplines and specialities [47].

Finally, there has been an increase in the implementation of MDT in SSA countries, and its efficacy is significant in improving the quality of care received by patients. However, factors such as poor infrastructure, which filter down to every aspect of society, act as a barrier to the proper implementation of MDT in these settings. Resources in the form of trained clinicians, equipment, and government support for patients still hinder the true efficacy of MDTs in SSA.

Awareness of multidisciplinary teams in Africa among healthcare professionals and the public

It is important to review the awareness and access to MDTs in the SSA countries. The MDT is a manifestation of the efficacy of teamwork between different healthcare providers as well as between the professions that make up the bulk of clinical care. Hence, attitudes toward teamwork in the clinical context are a proxy for attitudes toward MDT. A study from in the Ashanti region in Ghana showed that healthcare professionals spanning several specialities and roles generally agreed that teamwork improves the quality of care that patients receive [48]. Overall, 95% of the participants in the survey agreed/strongly agreed that “the team approach improves the quality of care to patients” [48]. However, the survey also highlighted that, although healthcare professionals are generally in favor of team-based care for patients, they are less in agreement about the implementation of such systems. Moreover, 70.1% of healthcare professionals strongly agreed/agreed and were neutral that “Developing an interdisciplinary patient care plan is excessively time-consuming,” with 27.6% of healthcare professionals being in the agreed/strongly agreed group [48]. The fact that such a significant number of healthcare providers did not disagree with this statement suggests that healthcare professionals in these settings view interdisciplinary care as one that is required but not attainable. This view may be a symptom of poor access to resources, in the form of staff members, equipment, and general infrastructure, such as electricity, in such resource-poor settings.

A prospective analysis of hospitals in SSA using data retrieved from a multi-center international collation of global surgical cancer management showed that an MDT approach was absent in 42.4% of hospitals studied [49]. These hospitals were selected based on the ability to provide elective or emergency breast cancer surgery and included South Africa, Ghana, Nigeria, and Kenya [49]. This study also found that only 55.3% of hospitals had access to a full-time pathologist, implying that it may be possible for hospitals that follow the MDT approach to not have access to a full-time pathologist [49]. This may then confer less efficacy of the MDT in providing care for patients compared to the canonical MDT model. Additionally, the study found that hospitals that followed an MDT approach, core members of staff were frequently missing, such as nurse oncology specialists, palliative care specialists, and oncologists [49].

A study examining the use of clinical guidelines and engagement with MDT in Nigeria found that only 38% of the clinicians whose roles are deemed core to MDT discussions routinely engaged with MDT discussions [50]. The study focused on healthcare professionals whose roles are important components of breast cancer MDT, namely, breast surgeons, clinical oncologists, radiologists, and pathologists [50]. This is the case despite 83% of the same set of clinical practitioners believing that access to regular MDT discussions confers better adherence with breast cancer guidelines and subsequently better care for patients [50]. This suggests that clinical practitioners involved in the care of breast cancer patients are generally aware of MDTs and the value that they provide to patients. However, due to poor resources, cultural norms (possible thoughts of implementation being too time-consuming), and poor infrastructure, this awareness is not translated into reliably functioning MDTs.

The relationship between cancer patients in SSA and the MDT system is difficult to unveil, as there is sparse literature directly studying the awareness of the populace with MDT. However, one factor consistently arises in the context of low-resource settings, especially in SSA, i.e., the out-of-pocket cost of cancer treatment [51]. According to a prospective study analyzing breast cancer patients in a public tertiary center in Nigeria, 70% of patients with breast cancer experience catastrophic healthcare expenditure due to out-of-pocket costs of their care [51]. Overall, 90% of women spend more than 40% of their annual family income on their care [51]. This leads to the inability to receive complete cancer care in the form of evidence-based adjuncts such as radiotherapy [51]. It also often leads to patients dropping out of anti-cancer regimes due to financial difficulty. This affects the relationship between MDTs and patients because even if a meeting is held, and a guideline-based treatment approach is selected, it is likely that the true efficacy of the MDT model is not projected through due to incomplete care [51,52].

Case studies of the establishment of multidisciplinary teams

There are several evidence-driven and subsequently well-established cancer MDTs for a variety of cancers in Africa. Some of the major studies are outlined below.

Cancer Multidisciplinary Teams in East Africa

Mozambique, a relatively low-income and GDP per-capita country when compared to other African counterparts, had its first MDT implementation in 2016 for breast cancer [17], which included international surgeons, oncologists, pathologists, radiologists, and radiation oncologists from who met weekly in line with the frequency and core members of the MDT seen in Western countries, but lacking palliative and specialist nurse input. The impact this had was reflected in greater follow-up visit frequency from three to four per year and subsequent enhanced continuity of care. This then likely translated to a three-year survival increase from 48% to 73% among early-stage disease patients and a 53% reduction in mortality, which is substantial, especially given the fact that it costed $802.96 per quality-adjusted life year gained, which was cost-effective even for a relatively deprived African country [17]. The statistics likely stem from more accurate and earlier staging, better and greater effective use of neoadjuvant chemotherapy, cleaner surgical margins, and better collaboration between medical and surgical aspects of cancer care [17].

Ethiopia, similar to Mozambique in eastern Africa, although at a much higher GDP per capita, has also demonstrated the impact of MDT on cancer management with colorectal cancer in the main tertiary cancer center in the country [11]. MDT was implemented in 2014, the first of its kind in the country, which involved surgeons (both lower and upper GI), oncologists, radiologists, and surgical residents. In 2016, a coordinator was established to manage referrals, circulate cases, and record outcomes. Consequently, 98% of treatment-naive patients who were discussed at the MDT had complete clinical staging compared to 52% who had treatment before MDT discussion [11]. This likely explains why, in this study, postoperative local recurrence was reported as 45% in the group surgically treated before MDT discussion, indicating blind and inadequate treatment plans without accurate staging information, going against guidelines [11].

Oncologic Multidisciplinary Teams in Southern Africa

Botswana, a country in southern Africa with an even lower GDP per capita than Mozambique, has successfully implemented MDT for cervical cancer. In Botswana, cervical cancer is the primary cause of cancer death in women, with 70% of women diagnosed with cervical cancer having co-existent HIV infection [53]. For cervical cancer, radiotherapy is integral to a disease-free state; however, in Gaborone, the capital of Botswana, there is only one unit that offers this crucial treatment [54]. The year 2015 saw the implementation of the first gynecological oncology MDT at Princess Marina Hospital, which covered patients from this hospital and the Gaborone private hospital, both in the capital [54]. The MDT involved a gynecologist, oncologists, pathologists, a palliative care specialist, and a nurse coordinator [20]. The median travel time for the patient was two hours. The odds of presenting with stage 4 disease increased for patients travelling three to five hours, demonstrating the devastating impact of having a scarce MDT referral center and how it can lead to late presentation, where treatment options are limited [54]. However, greater distance from the city likely equates to village communities where lack of access to cervical cancer detection leads to a greater stage of disease at presentation. This is backed up by data not being linear to travel time, implying that other factors are at play, for example, differences in socioeconomic status and health literacy [54]. For example, patients living further away were more likely to have an HIV infection [54]. Nonetheless, 62% of patients required only a single visit, and such efficiency can have huge financial benefits for such patients in village communities, as it means less time off work and reduced transportation costs [20]. Because of treatment formulation in fewer visits, this allowed treatment to start quicker, for example, in this case, the median time from biopsy to initiation of radiation therapy reduced from 108 to 39 days [20].

Cancer Multidisciplinary Teams in West Africa

Nigeria, one of the more developed countries in Africa, has demonstrated through two case studies the effect of MDT management of atypical cases of gastric cancer, especially the impact of a lack of cohesion between medical oncologists and surgeons to formulate an optimal management plan [55]. One case involved a 28-year-old gentleman with a three-week history of an acute abdomen associated with abdominal swelling. The patient was worked up for a perforation; however, ongoing symptoms prompted an MDT meeting at the bedside, leading to the diagnosis of gastric malignancy with further multidisciplinary agreement to start cytotoxic chemotherapy. Unfortunately, the patient passed away two weeks after decisions were made, and although an earlier diagnosis likely would not have changed the prognosis, it had a significant impact on the psychosocial journey of the patient in the last weeks of life [55].

The second case described in this Nigerian study was that of a 53-year-old gentleman who presented with a one-week history of malena, epigastric pain, and fainting. He was diagnosed initially with non-steroidal anti-inflammatory drug-induced erosive gastritis; however, blood workup led to a bone marrow aspirate, which showed signs of cancer, and CT noted widespread osteolytic bone lesions [55]. MDT meetings involving hematologists and orthopedics led to the diagnosis of disseminated intravascular coagulation from an unknown cancer, which was managed conservatively. MRI later showed metastatic vertebral disease with cord compression. The patient died three weeks after seeking care in the United States. This case again emphasizes how earlier MDT discussion, although it may not have changed prognosis, leads to better diagnostic accuracy as well as avoiding unnecessary investigations that patients may not benefit from. For example, endoscopy in the case of this patient, especially in countries like Nigeria where the necessary healthcare may not be free [55].

Cross-continental Oncologic Multidisciplinary Teams

A more novel MDT approach, which crosses borders, can be demonstrated by the Edinburgh-Malawi partnership [21]. Malawi is a low-income East African country where 50% of the population lives in poverty [56]. Healthcare professionals in this country are scarce, with just two oncologists in the entire country and no access to radiotherapy [21]. Queen Elizabeth Central Hospital (QECH) is the largest hospital in Malawi, and in association with Edinburgh Cancer Centre, United Kingdom, in 2012 formed MDTs which met monthly to discuss and make treatment decisions on all complex patients with cancer. This included breast cancer international video-linked collaboration in 2014, conducted in Malawi with oncology specialists from Boston, United States, with the aim of knowledge transfer and coaching to improve outcomes from breast cancer in patients from Malawi. This led to the development of MDTs for patients with breast cancer in QECH, which then paved the way for further weekly MDTs at QECH in a variety of specialities, ranging from head and neck cancers to sarcoma. Such collaborative work opened the gates for regular email discussion of patient cases in real time between MDT meetings [21].

A similar international approach seems to be occurring in Ghana, which has struggled with shortages of oncologists. Although there is no literature evidence of MDT implementation yet for cancer management, there is evidence for its use in surgical planning for complex oncological plastic reconstruction cases [57]. Kumasi, a city in Ghana, joined forces with the City Cancer Challenge in 2018 with a focus on strengthening MDTs and developing guidelines for management of common cancers [18]. With the help of the Mayo Clinic, a two-week training program was developed to build MDT capacity at Komfo Anokye Teaching Hospital in Kumasi, which occurred in 2021, from professionals spanning various specialities who would be expected to sit in a cancer MDT. This helped specialists see the impact of MDT on cancer care and helped build local treatment protocols. Overall, 90% of participants reported improved understanding of MDT structures and case management, and further, participants from the Mayo Clinic expressed interest in future collaborations [18]. The outcome of the initiative is a virtual learning platform now serving over 300 professionals in Africa, where there is weekly case-centric discussion of cases with MDT emphasis on management. Rather than demonstrating the impact of MDTs across borders, as with the Edinburgh-Malawi partnership, this study demonstrates the impact of short-term partnerships to facilitate the building of MDT infrastructure in African countries [18,21].

Implications for policy and practice

The establishment of MDT in cancer care in Africa is crucial to improving patient outcomes, enhancing care coordination, and addressing the continent’s rising cancer burden [47,58]. Key strategies to promote and support the establishment of MDTs in cancer care include policy development and government support, capacity-building and training, strengthening of healthcare infrastructure, collaboration and stakeholder engagement, and effective research with data collection [52].

To establish MDT in cancer care, African governments should create policies that mandate MDT in cancer care, ensuring they are part of national cancer control plans [59,60]. In addition to this strategy, African governments should increase funding for cancer care to include the training of healthcare workers in team-based approaches [60]. To guide funds and ensure that policies are adhered to, regulatory frameworks should be established in the form of guidelines mandating hospitals and cancer centers to implement MDT models for better patient management [60]. Additionally, African governments should encourage the establishment of regional cancer centers where MDT can operate efficiently. With existing regional cancer centers, improved access to diagnostic and treatment services can easily be established [5,61].

In the United Kingdom, the Expert Advisory Group on Cancer Treatment recommends the establishment of regional cancer centers whose role is to provide specialized care in terms of training, caseload, and MDT formation with well-established centers in breast and ovarian cancers [61].

To evaluate the effectiveness of cancer MDT, studies should be conducted periodically to assess their impact on patient outcomes, survival rates, and healthcare costs. National cancer registries should be provided to ensure accurate data on cancer trends and guide MDT policy making [62]. Education and training can also play a critical role in establishing and strengthening MDT for cancer care in Africa. Given the continent’s challenges of limited oncology specialists, inadequate healthcare infrastructure, and a fragmented cancer care system, a well-trained workforce is essential for effective MDT implementation [63]. One key strategy through which education and training can promote cancer MDT in Africa is through capacity-building for healthcare professionals [63,64].

Africa faces a severe shortage of oncologists and cancer specialists to address the continent’s cancer burden [63]. MDT training can empower general practitioners, nurses, and other health workers to take an active role in cancer management [64]. Training programs should focus on interdisciplinary collaboration, ensuring that all team members understand their role in the patient care pathway. Medical education should be mandated by African governments to incorporate MDT training in medical and nursing school curricula to create a culture of collaboration [65]. Continuous Professional Development (CPD) should involve workshops and training programs on MDT approaches for healthcare workers [66].

Furthermore, integration of MDT training in medical and nursing schools can help promote the MDT approach in healthcare [65]. To foster good outcomes, training should emphasize case-based learning, where students and trainees work through real-life cancer cases in MDT settings [65,66]. While the aforementioned strategies can promote capacity-building in cancer care MDT, well-structured CPD programs and on-the-job training can help sustain the MDT cancer care programs [67]. Regular CPD programs, workshops, and mentorship initiatives can enhance the skills of practicing oncologists, nurses, and other healthcare providers in MDT-based cancer care [65,66]. Hospital-based training programs should promote MDT meetings, allowing healthcare workers to gain firsthand experience in team decision-making [68].

In summary, education and training are the key strategies for promoting the establishment of MDT in cancer care across Africa. By integrating MDT education into medical curricula, investing in CPD, and strengthening teamwork skills, Africa can develop and sustain a skilled workforce that ensures effective, patient-centered, and collaborative cancer care.

Future research directions

There are various gaps in cancer MDT in Africa. First, the resources available for the control of cancer in Africa are not adequate, and the reasons for this are multifaceted and elaborate [63]. A lack of recognition of the disease in large sections of the population, with the absence of reliable national statistical data on cancer, coupled with insufficient advocacy, has severely limited meaningful political action to combat the disease [63]. In addition, the majority of African countries lack the necessary infrastructure and resources. The scarcity of a framework for cooperation in dealing with non-communicable diseases is also a barrier to dealing with cancer in these countries [69]. There is an acute shortage of skilled manpower for cancer care, with a very high patient-to-cancer specialist ratio, with few or no specialists in non-urban communities [69,70]. There is an unavailability of a well-coordinated supply chain for drugs, in addition to inadequate infrastructure across the states [70].

Concerning communication, non-specialist nurses need to learn practical communication skills when caring for patients diagnosed with cancer [71]. Effective communication in healthcare fosters hope, peace of mind, and trust in providers. Conversely, poor communication can result in subpar cancer care, heightened distress for patients and caregivers, and erosion of trust in healthcare providers [71]. Moreover, there is a lack of proper data collection and analysis. Establishing and maintaining cancer registries is crucial, warranting sustained investment in the African region to inform effective cancer control strategies [72]. Low funding and sustainability are other major setbacks in the establishment of cancer MDT [52]. Africa’s surging cancer cases are often diagnosed at late stages, reducing treatment success. One of the key factors contributing to this trend is financial constraints [52]. MDTs are acknowledged as the optimal framework for cancer care delivery, representing the gold standard in comprehensive and coordinated oncological management.

Regarding the aforementioned challenges, there are some potential future research directions. A probe into the role of technology in enhancing cancer MDT involving the use of computerized clinical decision-support systems (CDSS) was developed to help improve the quality of care and enhance decision-making [73]. Accordingly, CDSS possess significant potential to enhance the consistency and guideline concordance of MDT management recommendations by helping to gather detailed information for case deliberation, pointing out current evidence-based practices, and providing data on preceding management decisions for analogous cases [73]. Artificial intelligence (AI) has a key role in cancer MDT in Africa. It could help in many ways, which encompasses the ease with which information is gathered, analyzed, and provides possible solutions for improved patient outcomes in Africa. AI can find new biomarkers from data to help in tumor screening, detection, diagnosis, management, and prognosis prediction, thereby providing the best treatment for individual patients and improving their clinical outcome [74,75].

Future research should review, identify, and address barriers to MDT implementation in Africa, such as limited resources, inadequate infrastructure, and a lack of skilled manpower. A multi-institutional study of barriers to cervical cancer care in SSA by Kambhampati et al. cited financial burden in terms of the cost of treatment and transport expenses. This was attributed to the fact that the treatment cost was an out-of-pocket system and not universalized by the government [76]. Improved funding and support by the government will play a major role in improving future research directions concerning cancer MDT in Africa. The aim of future research should be to develop cancer MDT models that are directed toward the specific needs and contexts of African countries. The majority of barriers in African countries are a lack of infrastructure, skilled professionals, and funding. Future research needs to develop models that will address the above-mentioned issues in African countries. Strategies, for example, making provision for the services of a specialist closer to patients, might improve access to prompt care and compliance with management advice [77].

Several types of studies have the potential to significantly impact the efficacy of cancer MDT and patient outcomes in Africa. Observational studies, including retrospective and prospective cohort studies, can provide valuable insights into factors associated with improved patient outcomes. Retrospective cohort studies can analyze existing data to identify patterns and correlations, while prospective cohort studies can track patients over time to assess the impact of cancer MDT on their outcomes. In addition to observational studies, interventional studies such as randomized controlled trials can evaluate the effectiveness of specific interventions, including training programs and care pathways, on cancer MDT efficacy and patient outcomes. These studies can help determine the most effective strategies for improving cancer care in Africa.

Furthermore, systematic reviews and meta-analyses can synthesize evidence from multiple studies to assess the effectiveness of cancer MDT in African settings. By quantitatively combining data from various studies, meta-analyses can estimate the overall impact of cancer MDT on patient outcomes, providing a comprehensive understanding of what works best in these contexts. These studies can ultimately inform strategies to enhance cancer MDT efficacy and improve patient outcomes in Africa.

## Conclusions

Cancer is at the forefront of modern-day non-communicable diseases. A substantial proportion of the patients suffering reside in Africa, and, unfortunately, many of them are not well managed. Lack of proper MDT implementation or lack thereof, a cornerstone to cancer care, markedly underlies this suboptimal management. This can be attributed to several reasons, primarily fragmented care systems, insufficient infrastructure in human resources and technology, and lack of government involvement and funding. Proper and widespread MDT implementation in African nations can be afforded with integration into medical education, more longitudinal studies on the efficacy of MDT cancer-based care, with subsequent presentation, and involvement of key stakeholders, including the government.
